# Negative Feedbacks on Bark Beetle Outbreaks: Widespread and Severe Spruce Beetle Infestation Restricts Subsequent Infestation

**DOI:** 10.1371/journal.pone.0127975

**Published:** 2015-05-22

**Authors:** Sarah J. Hart, Thomas T. Veblen, Nathan Mietkiewicz, Dominik Kulakowski

**Affiliations:** 1 Department of Geography, University of Colorado, Boulder, Colorado, United States of America; 2 School of Geography, Clark University, Worcester, Massachusetts, United States of America; University of Nevada Reno, UNITED STATES

## Abstract

Understanding disturbance interactions and their ecological consequences remains a major challenge for research on the response of forests to a changing climate. When, where, and how one disturbance may alter the severity, extent, or occurrence probability of a subsequent disturbance is encapsulated by the concept of *linked disturbances*. Here, we evaluated 1) how climate and forest habitat variables, including disturbance history, interact to drive 2000s spruce beetle (*Dendroctonus rufipennis*) infestation of Engelmann spruce (*Picea engelmannii*) across the Southern Rocky Mountains; and 2) how previous spruce beetle infestation affects subsequent infestation across the Flat Tops Wilderness in northwestern Colorado, which experienced a severe landscape-scale spruce beetle infestation in the 1940s. We hypothesized that drought and warm temperatures would promote infestation, whereas small diameter and non-host trees, which may reflect past disturbance by spruce beetles, would inhibit infestation. Across the Southern Rocky Mountains, we found that climate and forest structure interacted to drive the 2000s infestation. Within the Flat Tops study area we found that stands infested in the 1940s were composed of higher proportions of small diameter and non-host trees ca. 60 years later. In this area, the 2000s infestation was constrained by a paucity of large diameter host trees (> 23 cm at diameter breast height), not climate. This suggests that there has not been sufficient time for trees to grow large enough to become susceptible to infestation. Concordantly, we found no overlap between areas affected by the 1940s infestation and the current infestation. These results show a severe spruce beetle infestation, which results in the depletion of susceptible hosts, can create a landscape template reducing the potential for future infestations.

## Introduction

In the context of a changing climate and increases in forest disturbances such as bark beetle infestations and wildfires, disturbance interactions are receiving increased attention in ecological research [1,2]. In particular, there is a need to better understand when, where and how one disturbance event may alter the severity, extent, or probability of occurrence of a subsequent disturbance, a concept known as *linked disturbances* [3]. A prior disturbance may amplify the second by increasing its likelihood or severity through positive feedbacks (e.g. blowdowns may increase the amount breeding material thereby increasing insect populations and likelihood of outbreak [4]). Or, alternatively the first disturbance may dampen the probability of occurrence or severity of the second (e.g. stand-replacing fire may decrease the probability of subsequent fire [5]).

During the late 20^th^ and early 21^st^ century, warm and dry conditions and suitable hosts have promoted landscape-scale (sensu [6]) and severe bark beetle outbreaks, resulting in tree mortality across 8.4 ± 2.5 Mha in the western North America (1997–2010; [7]). Given this extensive tree mortality, there is an increased need for understanding how bark beetle infestations alter subsequent disturbance dynamics. Considerable research has emphasized the potential effects of bark beetle outbreak on the fire behavior [3,8–14], occurrence [15–19], and severity [15,20–22]. Recent research has also emphasized the compound effects of bark beetle outbreak and fire on ecosystem recovery [20–23]. Far less is understood about how one bark beetle outbreak affects a subsequent outbreak.

Bark beetles of the *Dendroctonus* genus inhabit the inner bark and feed on the tree’s phloem tissues. Heavy colonization and reproduction within the inner bark interrupts the flow of water and nutrients throughout the tree and usually causes tree death. When and where bark beetle outbreaks occur is constrained by both weather and forest structure conditions [6,24,25]. Warm temperatures promote the rapid growth of beetle populations by increasing the proportion of beetles that develop within one year and decreasing overwintering mortality [26–28]. Drought may stress host trees, increasing the susceptibility of trees to infestation [29–32]. Forest structure also affects the occurrence of bark beetle infestations. Bark beetles prefer large diameter trees, growing in dense stands composed predominantly of the host tree species [6,33].

In the Southern Rocky Mountains, outbreaks of spruce beetles (*Dendroctonus rufipennis*) are among the most important broad-scale disturbances in subalpine forests. Spruce beetles are found in Engelmann spruce (*Picea engelmannii*) and subalpine fir (*Abies lasiocarpa*) forests, where they most frequently colonize large diameter (> 23 cm diameter at breast height; DBH) spruce trees. However when beetle population levels are high and host trees are severely-drought stressed, spruce beetles may attack trees less than 10 cm DBH [34]. Like other bark beetles, heavy colonization and reproduction within the inner bark usually kills the host tree. In northwestern Colorado, severe spruce beetle infestations tend to occur at median intervals of c. 70 years for the same stand [30,35]. The return interval of spruce beetle infestations to the same stand or relatively homogeneous landscape is hypothesized to be in part a function of a negative linkage between infestations. Thus, for forest stands (100s of hectares) and forest landscapes (1000s to tens of 1000s) that are characterized by similar forest compositions and tree population age structures, forest attributes are likely to affect the probability of occurrence and severity of an outbreak [32]. For example, a severe spruce beetle outbreak, which may result in the mortality of 90% of the mature host trees (Engelmann spruce), has been hypothesized to decrease the likelihood of subsequent infestation [33]. This decrease in susceptibility to infestation is hypothesized to persist until host trees reach a suitable size for infestation. While there are studies documenting the collapse of an outbreak evidently due to host depletion [29], there is no published empirical evidence for a bark beetle infestation negatively influencing the occurrence of a subsequent bark beetle infestation.

**Fig 1:**
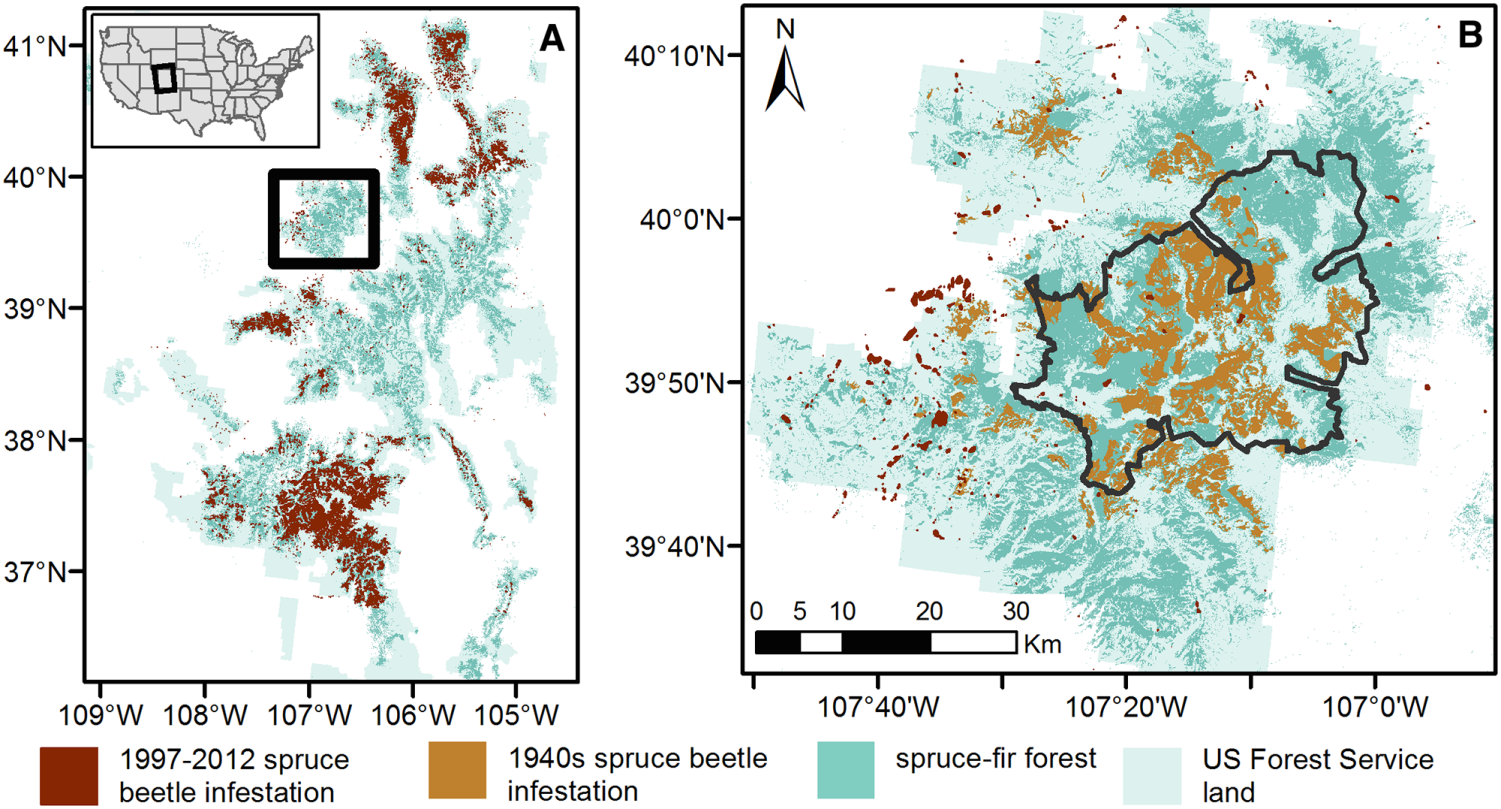
The larger study region and study area. (A) Map of the Southern Rocky Mountain study region displaying spruce-fir forests infested by spruce beetles during the 1997–2012 period. The upper left inset displays the location of the study region in relation to the entire United States. The black box indicates the study area displayed in B. (B) Map of the Flat Tops study area comprised of the Flat Tops Wilderness (black line) and adjacent areas of White River National Forest and areas infested by spruce beetles during the 1940s and 1997–2012 periods. Sources are given in Table 1.

A widespread spruce beetle outbreak affected a large part of the spruce-fir forests of western Colorado in the 1940s. This outbreak was most severe in the Flat Tops Wilderness area of White River National Forest in northwestern Colorado where 99% of the overstory spruce were killed over an area of 2,700 km^2^ [33,36]. The second most severely affected area in the 1940s outbreak was Grand Mesa National Forest to the southwest of White River National Forest where mortality was estimated at over 50% [32]. There are no other known 20^th^ century spruce beetle outbreaks in Colorado of a comparable magnitude to the 1940s outbreak that was centered on the Flat Tops area of White River National Forest. Thus, in the context of the recent spruce beetle outbreak of 1997–2012, the concentration of high tree mortality during the 1940s outbreak in one large contiguous area created the opportunity to quantitatively evaluate the potential for a landscape-scale bark beetle infestation to negatively affect the probability of a subsequent infestation ca. 60 years later. Mapping of the recent spruce beetle outbreak from Aerial Detection Surveys [37] indicate a low spruce beetle infestation in the Flat Tops area in comparison with spruce-fir forests throughout Colorado (Fig 1). Thus, the primary aim of this study is to determine if the relative lack of recent spruce beetle infestation in the Flat Tops area is due to a negative feedback from host depletion attributable to the 1940s outbreak. Because spruce beetle infestation depends on both climate and forest conditions [6], we first assess the suitability of climate and forest attributes for spruce beetle infestation during 1997–2012 in the Flat Tops study area in comparison with the entire Southern Rocky Mountain Ecoregion of the U.S. Second, we examine forest attributes across the Flat Tops study area in relation to the mapped extent of the 1940s spruce beetle infestation and compare current forest structure in field sampled stands infested and not infested in the 1940s. Thus, by documenting the climatic suitability of the Flat Tops study area for the recent infestation, we are able to associate the relative absence of infestation with host depletion from the 1940s outbreak.

## Materials and Methods

### Study area

The study region (Fig 1) is the spruce-fir forest type of the Southern Rocky Mountain Ecoregion. The study region is characterized by high elevations (3215 ± 205 m), cold, wet winters (mean minimum January temperature -14°C and mean total January-March precipitation 241 mm; 1981–2010) and warm, dry summers (mean maximum July temperature 20.6°C and mean total June-August precipitation 169 mm; 1981–2010) [38]. Engelmann spruce and subalpine fir co-dominate the spruce-fir forest type.

We examine the potential for spruce beetle infestation to affect the area of forest structure suitable for subsequent spruce beetle infestation within a subset of the study region comprised of the Flat Tops Wilderness and adjacent areas of White River National Forest of northwestern Colorado, USA (Fig 1). The Flat Tops study area was chosen because of the unique availability of maps of both the 1940s spruce beetle infestation derived from air photo interpretation [5] and the current (1997–2012) spruce beetle infestation produced from Aerial Detection Surveys (ADS; [37]). Historical reports document widespread spruce beetle infestation in the 1940s, when about 25% of the merchantable volume of Colorado’s spruce was killed. The Flat Tops study area experienced particularly abundant mortality, characterized by more than 90% canopy mortality [33].

### Data processing

We first obtained data on the occurrence of spruce-fir forest across the Southern Rocky Mountain study region (Table 1). Most vegetation cover-type datasets express only moderate (40–60%) overall agreement between field plot data and forest cover-type at 30 x 30 m spatial scale [39,40], thus we combined three datasets depicting the occurrence of spruce-fir forest [41]. For each vegetation dataset, we listed the presence of a spruce beetle host within a 990 x 990 m pixel, which approximates a stand scale [41]. We adopted a conservative criterion for mapping spruce-fir forest based on requiring its presence in all three datasets.

**Table 1:** The GIS data layers and attributes used to examine linked spruce beetle disturbance.

Variable	Description	Data	Type	Resolution	Year
Damage casual agent	Name of forest pest or pathogen causing damage	Aerial Detection Survey Database [37]	Polygon	Compiled at 1:100,000 scale	1998–2013
1940s infestation	Presence /absence of 1940s spruce beetle infestation	Bebi et al. 2003 [5]	Polygon	Interpreted at 1:10,000 scale	Based on 1971 color & 1984 IR aerial imagery
R2VEG Cover type	Dominant life forms, based on Society of American Foresters classification	R2VEG [42]	Polygon	Interpreted at 1:24,000 scale	Based on 2002 aerial imagery
LANDFIRE EVT	Existing vegetation type, based on Nature Serve’s ecological systems classification	LANDFIRE [43]	Raster	30 x 30 m	Based on 2001–2010 Landsat imagery
GAP Analysis Project Cover type	Primary cover type	GAP	Polygon	Interpreted at 1:100,000 scale	Based on 1989–1998 Landsat imagery
R2VEG Diameter at breast height	Tree DBH binned (cm): 1) <2.5, 2) 2.5–12.4, 3) 12.5–22.9, 4) 23–40.4, 5) ≥40.5	R2VEG [42]	Polygon	Interpreted at 1:24,000 scale	Based on 2002 aerial imagery
Southern Rocky Mountain Ecoregion	Level III Ecoregions	North America Ecoregions [44]	Polygon	Compiled at 1:250,000 scale	2013
August maximum temp	average monthly maximum temperature (°C)	PRISM [38]	Raster	4 x 4 km	1997–2012
Annual precipitation	average annual precipitation (mm)	PRISM [38]	Raster	4 x 4 km	1997–2012
March minimum temperature	average monthly minimum temperature (°C)	PRISM [38]	Raster	4 x 4 km	1997–2012
October minimum temperature	average monthly minimum temperature (°C)	PRISM [38]	Raster	4 x 4 km	1997–2012

Next we obtained spatially explicit data on the presence of spruce beetle infestation over the time period from 1998–2013 from the United States Forest Service Region 2 ADS database [37]. Aerial Detection Surveys have been conducted annually in the Southern Rocky Mountains since 1994. To our knowledge robust accuracy assessments of ADS maps of spruce beetle infestation do not exist. However, accuracy assessments between ADS and ground reference data listing the presence/absence of bark beetle infestation in lodgepole pine show moderate-high agreement at coarse (500 m) spatial grains [39,40]. Thus we assumed ADS maps of spruce beetle infestation are most appropriate for assessing coarse-grain trends in presence/absence of infestation. To account for the ca. 1-year lag between initial infestation and ADS detection, we shifted the year of detection back one year to obtain year of attack [7]. Annual spatial polygon data listing the year of spruce beetle attack (1997–2012) were then converted to a 990 x 990 m grid listing the presence of spruce beetle infestation. Annual grids were then summed to obtain the cumulative area infested (1997–2012) and multiplied by a raster of spruce-fir presence to obtain a cross-validated grid of spruce beetle infestation [24].

We also obtained a map of the presence of the 1940s infestation within the Flat Tops study area [5]. To our knowledge no other maps of the 1940s outbreak exist for the Southern Rocky Mountains. Maps of the 1940s infestation were developed from visual stereoscopic examination of 1971 color and 1984 IR aerial imagery (minimum mapping unit 5 ha). Stands mapped as infested by spruce beetles during the 1940s were defined as stands in which >30% of canopy trees were dead [5]. Spatial polygon data on the occurrence of the 1940s infestation was then converted to a 990 x 990 m grid listing the presence of spruce beetle infestation and multiplied by the raster of spruce-fir presence to obtain a cross-validated grid of spruce beetle infestation [24].

Finally we obtained spatial data on climate and forest structure variables, which were hypothesized to be important in predicting the occurrence of spruce beetle infestation. We obtained gridded monthly precipitation and temperature data from the Parameter-elevation Regressions on Independent Slopes Model (PRISM; [38]) (Table 1). To determine if warm and dry weather was associated with infestation, we calculated the 1997–2012 means of maximum August temperature and total annual precipitation, which previous research has shown to predict occurrence of spruce beetle infestations [25,29,45]. To determine if anomalously cold weather during the late autumn to early spring was associated with the presence/absence of infestation we calculated the 1997–2012 means of minimum October and March temperature, which are understood to inhibit infestation [25,45]. Next, we obtained vegetation layers depicting the mean diameter at breast height (DBH) size classes for the dominant canopy species, which were created from manual aerial photo interpretation of 1-m resolution color aerial photographs in 2002 (Table 1).

### Determining the biophysical drivers of spruce beetle infestation

We used two methods to assess the biophysical variables driving the spatial variability in the occurrence of 1997–2012 spruce beetle infestation in the Southern Rocky Mountain study region. First, we used a spatial overlay approach [46,47], where spatial data on spruce beetle infestation were compared with spatially explicit climate and forest structure data (Table 1). We used spatial overlays to calculate the conditional probability of the presence/absence of spruce beetle infestation given each value of the independent variable. Conditional probability is a measure of the probability of the dependent variable (presence or absence of spruce beetle infestation) occurring given each value of the independent variable (biophysical variables). Continuous climate variables were first binned into four equal-interval classes [48]. Then we tabulated the number of 990 x 990 m pixels of all values of each independent variable that occurred in uninfested and infested areas and calculated the conditional probability of infestation. The null hypothesis is that spruce beetle infestation is independent of all values of each independent variable and thus observed conditional probabilities of infestation should equal conditional probabilities of uninfested stands. Our spatial overlays assessed entire populations and not samples. Thus all deviations between conditional probabilities are viewed as real differences between the datasets and statistical tests are not necessary. However, given that our spatial datasets exhibit classification error, we conservatively assumed that only differences greater than 10% are meaningful (e.g. [46]).

Second, to complement our conditional probability analysis of univariate relationships between biophysical predictors and the presence/absence of spruce beetle infestation, we used a Conditional Inference Framework (CIF; [49]) to assess multivariate relationships. CIF is similar to Random Forests [50] in that many classification trees are constructed by dividing the data into increasingly homogenous groups based on splits in the independent variables [49,51,52]. Classification trees are useful for detecting nonlinear relationships and interactions between variables [51]. In contrast to Random Forests where variable selection is based on the maximization of an information criterion (e.g. Gini coefficient), CIF uses conditional permutation-based significance tests to select variables [49]. This decreases selection bias in cases where independent variables have substantially different numbers of potential splits (e.g. categorical vs. continuous independent variables) [53], or where independent variables are correlated [54]. To evaluate the variables most important for predicting the presence/absence of spruce beetle infestation, we calculated conditional variable importance scores, a measure of each independent variable’s contribution to overall model fit [54]. Because the calculation of conditional variable importance is computationally intensive, we randomly selected 2000 cases, stratified by spruce beetle infestation (1000 infested; 1000 uninfested). Model accuracy was asssed using overall accuracy and model sensitivity and specificity.

### Effects of the 1940s spruce beetle infestation on the 1997–2012 infestation

To determine if the effects of the 1940s spruce beetle infestation on forest structure may affect the susceptibility of a stand to subsequent infestation in 1997–2012, we first used our model of the presence/absence of spruce beetle infestation to determine the relative importance of forest structure versus climate variables in constraining infestation within the Flat Tops study area. We tabulated the number of pixels within each model node and evaluated the relative importance of splits in climate vs. forest structure variables in predisposing the Flat Tops study area to infestation in 1997–2012. To this end, we calculated the percent of pixels in each model node for the entire Southern Rocky Mountain Study region and just the Flat Tops study area (Southern Rocky Mountain Study Region % | Flat Tops study area %). If the percent of pixels that met the condition were greatly different (>10%) for the Flat Tops study area than for entire Southern Rocky Mountain Study region, then that condition was interpret to be disproportionately important in constraining/promoting infestation within the Flat Tops study area.

Next, we coupled fine-scale field data with stand-level spatial data to determine if forest structure was altered by previous spruce beetle infestation. First, to test if large trees were depleted in areas of the 1940s infestation, we tabulated the number of 990 x 990 m pixels of all values of tree size that occurred in areas with and without 1940s infestation [55]. Then we calculated the conditional probability of the dominant tree size class (2.5–12.4, 12.5–22.9, 23–40.4, or ≥40.5 cm DBH) given the presence/absence of 1940s infestation.

Because the available GIS dataset depicting tree size is not species specific, we collected stand-scale (0.01 ha) field data to determine the delayed effects of a severe spruce beetle infestation on species composition. Field data were collected in the summer of 2013 at 7 sites (4 sites without evidence of 1940s infestation and 3 sites with evidence of severe spruce beetle infestation in the 1940s) across the Flat Tops study area. Plots were located using maps of the presence/absence of the 1940s infestation [5]. We field verified that our sites were located in areas affected by the 1940s infestation by locating large, dead, standing snags with spruce beetle galleries. At each site we collected data from a cluster of 10 randomly-located 100 m^2^ plots. For each tree in the plot, we recorded the species, the diameter at breast height (DBH), and tree status (live, dead, or fallen). We then aggregated data for stands that experienced and did not experience severe spruce beetle infestation in the 1940s and calculated the 2000s density of live spruce and fir. We then compared 2000s stand structure and composition in stands uninfested and infested during the 1940s.

Finally, we used spatial data to assess if these structural differences between stands uninfested and infested during the 1940s affected the distribution of 1997–2012 infestation within the Flat Tops. We overlaid a 990 x 990 m grid of 1997–2012 spruce beetle infestation presence/absence with a 990 x 990 m grid of 1940s infestation presence/absence and calculated the area of overlap.

## Results

### Biophysical drivers of the 1997–2012 spruce beetle infestation

Across the Southern Rocky Mountain study region, spruce beetles infested approximately 15% of the spruce-fir zone over the period from 1997–2012 (areas mapped as infested in ADS surveys 1998–2013; Fig 1A). Over this time period, the Flat Tops study area has experienced very little infestation (2% of the spruce fir-zone recorded presence of infestation; Fig 1). While the annual area infested by spruce beetles across the Southern Rocky Mountain study region has been growing since 1998 [55], ADS data indicate that most spruce beetle activity in the Flat Tops study area occurred prior to 2005 (S1 Fig).

**Fig 2:**
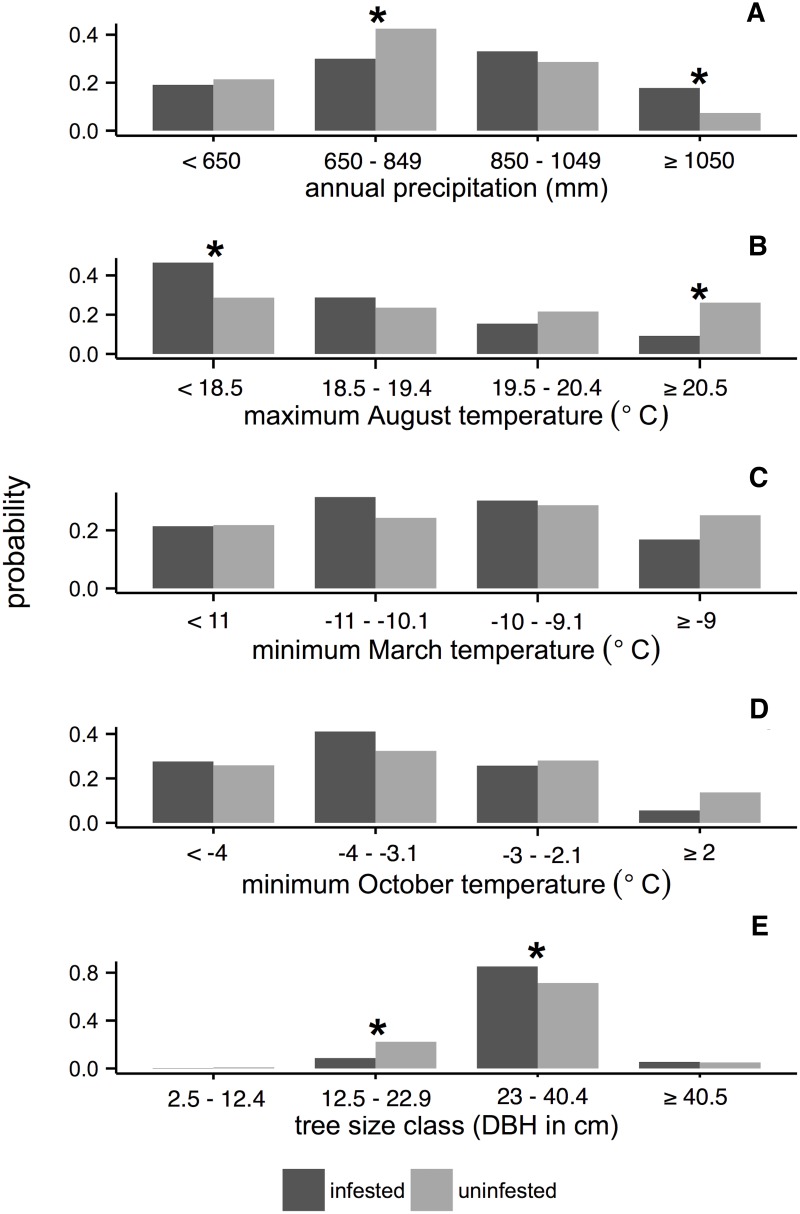
Conditional probabilities of the presence/absence of spruce beetle infestation (1997–2012) given selected bioclimatic variables in the Southern Rocky Mountains study region. (A) annual precipitation, (B) maximum August temperature, (C) minimum March temperature, (D) minimum October temperature, and (E) tree size class for uninfested and infested stands. Dark gray bars indicate conditional probability of spruce beetle infestation given that value of a bioclimate variable across the Southern Rocky Mountain study region. Light gray bars indicate the conditional probability of uninfested forest. The asterisk symbol (*) above a pair of bars indicates a meaningful difference between conditional probability of uninfested and infested forest (i.e. difference > 10%, see *Methods* for more description). Note y-axes extend over different ranges.

Across the Southern Rocky Mountain study region, spatial overlay analysis revealed meaningful differences between the conditional probabilities of uninfested and infested spruce-fir forest given climate and forest structure variables (Fig 2). Contrary to expectations, spruce beetle infestation was less likely in areas with high maximum August temperatures (≥19.5°C; Fig 2B). However this difference was only meaningful in areas where the average maximum August temperature was greater than ≥20.5°C. Areas with cooler maximum August temperatures (<18.5°C) were more likely to be infested. Also contrary to expectation, areas with high annual precipitation (≥ 1050 mm/year) were more likely to experience spruce beetle infestation, while areas with moderately low annual precipitation (650–849 mm/year) were less likely to experience infestation (Fig 2A). There were no meaningful differences between the probabilities of uninfested and infested forest given any of the four classes of minimum March temperature or minimum October temperature (Fig 2C and 2D).

We also found that forest structure differed between forests uninfested and infested by spruce beetles in 1997–2012 (Fig 2E). Spruce beetle infestation was more likely to occur in areas with large diameter trees (≥ 23 cm DBH; Fig 2E). For stands with smaller diameter trees (< 23 cm DBH), the probability of infestation was < 0.22 (Fig 2E).

**Fig 3:**
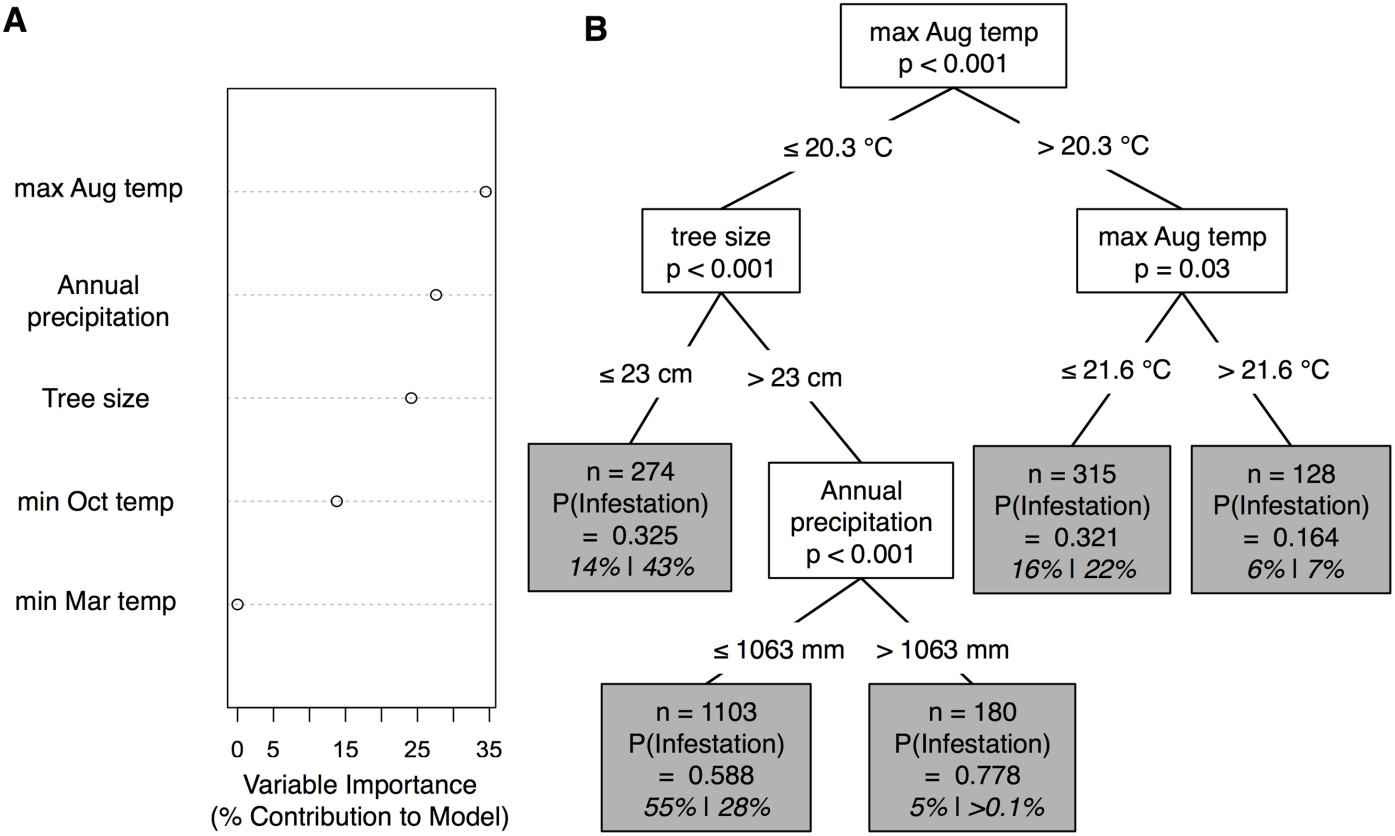
Results from conditional inference forest analysis of the presence/absence of spruce beetle infestation with climate and forest structure data in the Southern Rocky Mountain study region. (A) Conditional variable importance for the five biophysical variables used to model the occurrence of spruce beetle infestation across the Southern Rocky Mountain study region. Conditional variable importance scores were calculated following the Random Forest principle of mean decrease in accuracy and then transformed to express the contribution of each variable to the overall model. Higher values indicate variables are more important to the classification. Conditional variable importance scores represent 1000 model runs. All trees were built using a random sample of 2000 cases, stratified by the presence/absence of spruce beetle infestation (1000 infested and 1000 uninfested). Overall prediction accuracy is 81%. (B) A classification tree for determining the presence of spruce beetle infestation from uninfested spruce-fir stands across the Southern Rocky Mountains study region. On the tree, if condition is satisfied, proceed to the left of the tree. Tree nodes (gray boxes) describe the number of pixels across the entire Southern Rocky Mountain study region that meet the condition and the probability of spruce beetle infestation. The gray boxes also list the percent of pixels that meet the conditions for the entire Southern Rocky Mountain Study region and just the Flat Tops study area (Southern Rocky Mountain Study Region % | Flat Tops study area %). If the percent of pixels that meet the condition are greatly different (>10%) for the Flat Tops study area than for entire Southern Rocky Mountain Study region, then that condition is disproportionately important in constraining/promoting infestation within the Flat Tops study area.

The multivariate model of 2000s spruce beetle uninfested and infested forest performed reasonably well. The CIF model correctly predicted 809 of the 1000 pixels with spruce beetle infestation (i.e. sensitivity = 0.81), and correctly predicted 819 of the 1000 pixels without spruce beetle infestation (i.e., specificity = 0.82). Variables important in predicting 2000s spruce beetle infestation included maximum August temperature, annual precipitation, and tree size class (Fig 3A). Spruce beetle infestation was unlikely to occur in areas with maximum August temperatures above 20.3°C (probability of infestation = 0.276). Infestation was particularly unlikely when temperatures exceed 21.6°C (probability of infestation = 0.164; Fig 3B). However, more than 75% of the study area was characterized by 1997–2012 mean maximum August temperatures cooler than 20.3°C (S2 Fig). In these areas, spruce beetle infestation was particularly like to occur in areas with large trees (≥ 23 cm DBH; Fig 3A) and high precipitation (>1063 mm/year) (Fig 3B and S2 Fig).

### Effects of the 1940s spruce beetle infestation on the 1997–2012 infestation

Applying the decision tree to the pixels within the Flat Tops study area provided insight into the biophysical predictors important in constraining infestation in the Flat Tops study area. Across the Flat Top study area about 29% of pixels were characterized by 1997–2012 mean maximum August temperatures unsuitable for infestation (<20.3°C; Fig 3B and S2 Fig). An additional 43% of the pixels within the Flat Tops study area were characterized by small diameter trees (<23 cm DBH), which inhibit infestation (Fig 3B and S2 Fig). In comparison to the entire Southern Rocky Mountain study region, the percent of pixels with small diameter trees (<23 cm DBH) in the Flat Tops study area was three times greater (43% vs. 14%, for the Flat Tops study area and entire Southern Rocky Mountain study region, respectively; Fig 3B and S2 Fig). As a result, the percentage of pixels that were split based on annual precipitation was far lower for the Flat Tops study than the Southern Rocky Mountain study region.

**Fig 4:**
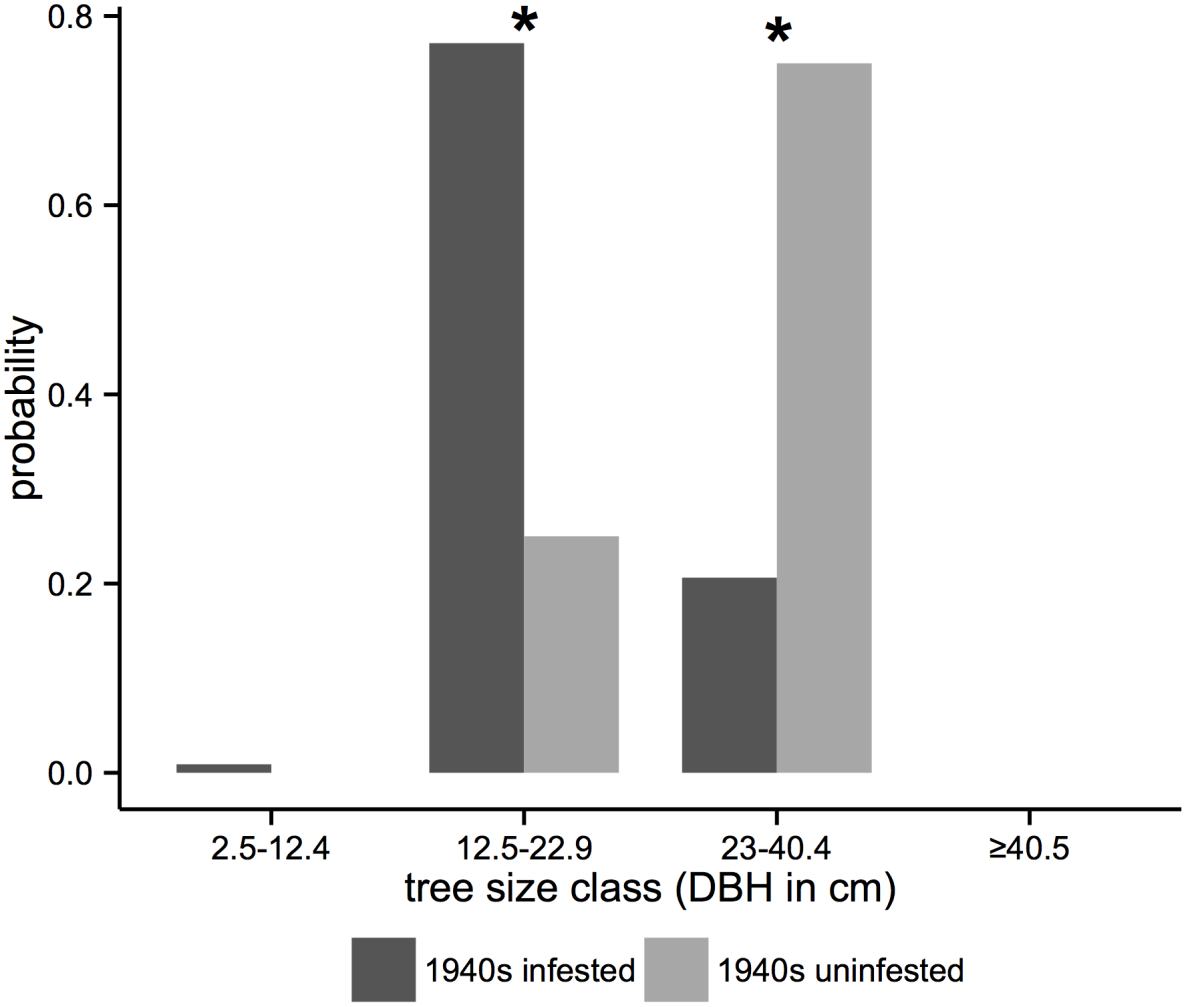
The conditional probability of current dominant tree size given the presence or absence of the 1940s spruce beetle infestation in the Flat Tops study area. Dark gray bars indicate the probability that a 990 x 990 m spruce-fir pixel is infested by spruce beetles; light gray bars indicate the probability a pixel is uninfested. The asterisk symbol (*) above a pair of bars indicates a meaningful difference between conditional probability of uninfested and infested forest.

**Fig 5:**
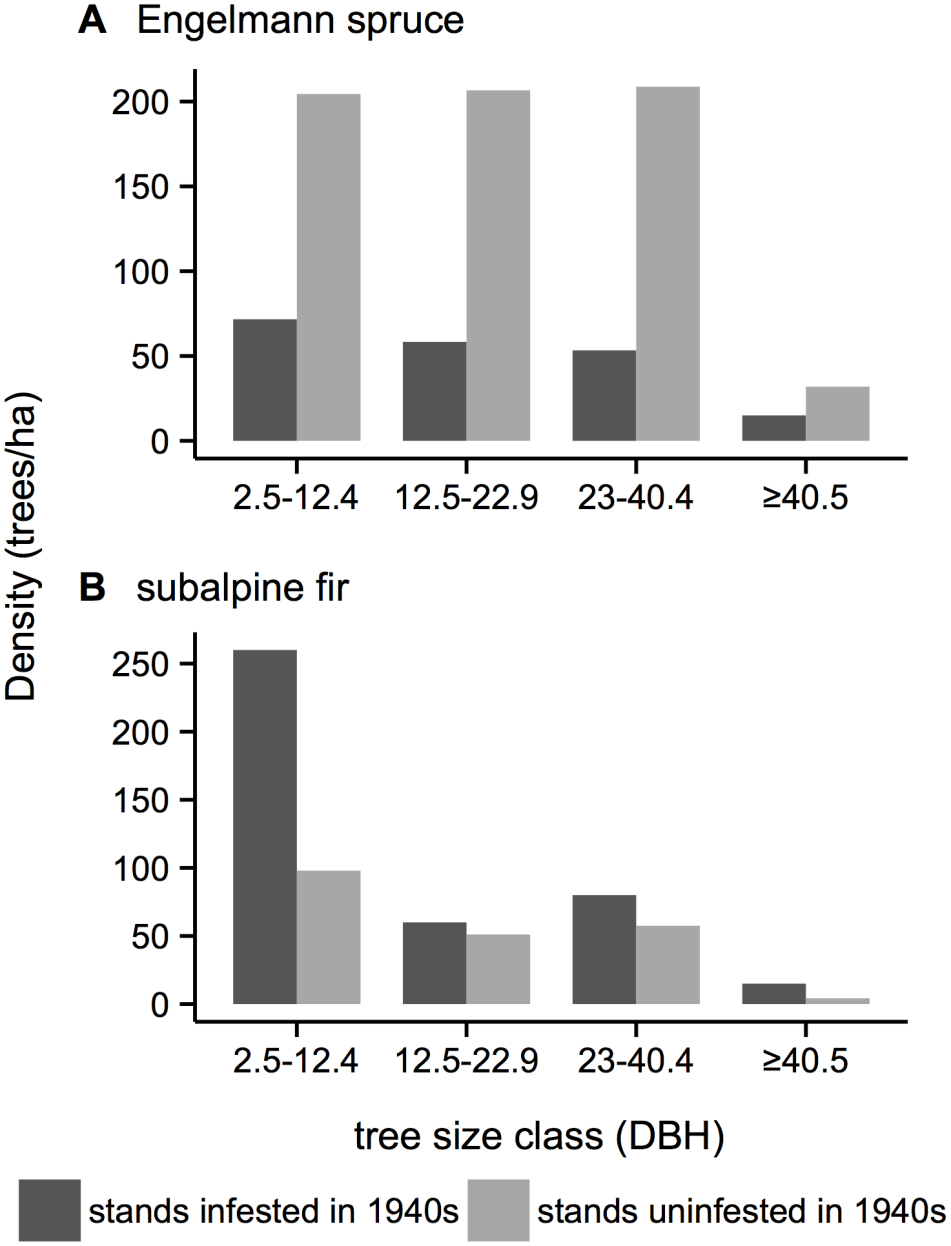
Current (2000s) tree size class distributions in stands uninfested and infested during the 1940s infestation within the Flat Tops and adjacent areas of White River National Forest. Data represent the aggregate of all plots (stands uninfested during the 1940s outbreak, n = 4 sites each with 10 ca. 100 m^2^ plots; stands infested during 1940s outbreak, n = 3 sites each with 10 ca. 100 m^2^ plots).

Within the Flat Tops study area, comparison of forest structure of 990 x 990 m in areas uninfested and infested by the 1940s infestation indicates that infested stands are characterized by smaller tree sizes (12.5–22.9 cm DBH) 60 years following infestation (Fig 4). This coarse-scale finding based on mapping from aerial photographs (Table 1) is supported by stand-level field measurements. During the 1997–2012 period of spruce beetle infestation, field data revealed that in comparison with stands infested during the 1940s, stands not infested in the 1940s had consistently higher densities of spruce in all size classes including the largest class (i.e. ≥ 40.5 cm DBH; Fig 5). In contrast, 60 years following the 1940s spruce beetle outbreak subalpine fir was more abundant in all size classes in stands infested during the 1940s outbreak compared to uninfested stands. Concordantly, we found no overlap between areas infested during the 1940s and the 2000s infestation (Fig 1A). Within the Flat Tops region only three 990 x 990 m pixels were infested in 1997–2012, but none of those overlapped with the 254 pixels infested in the 1940s. Instead, all pixels infested in 1997–2012 were located in areas with large diameter trees (≥ 23 cm DBH).

## Discussion

Across the Southern Rocky Mountain study area, spruce beetle infestation was more likely to occur in areas with cool to moderately warm mean maximum August temperatures and higher amounts of annual precipitation. Although these results at first glance seem counter-intuitive given the importance of drought in triggering spruce beetle outbreaks [28–30,56], our results are spatial associations of infestation with mean conditions rather than temporal associations with drought events measured as departures from longer-term average conditions. While bark beetles preferentially attack drought-stressed trees [28, 29], our results describe habitat suitability for spruce beetle, which clearly is greater at the cooler and wetter sites where spruce is more common. In contrast, warmer sites are likely to be characterized by greater proportions of non-host species (e.g. lodgepole pine) and provide less potential for spruce beetle outbreak. Overall, we interpret the association of spruce beetle infestation with cooler and wetter sites as being explained primarily by the greater presence of host species at those sites.

Across the Southern Rocky Mountain study area, spruce beetle infestations have occurred overwhelmingly in spruce-fir stands dominated by large trees (≥ 23 cm DBH). This corresponds with empirical results from Grand Mesa National Forest in western Colorado, which showed early 2000s spruce beetle infestation was significantly more likely in spruce larger than 24 cm DBH [57]. Spruce beetles prefer large trees, which provide both higher amounts of phloem for beetles to feed upon and thicker bark that increases overwinter survival rates [32].

While both climate and forest structure interacted to drive the occurrence of spruce beetle infestation across the Southern Rocky Mountains, our data suggest that the current infestation in the Flat Tops was severely constrained by a low proportion of large trees (≥ 23 cm DBH). Our model suggests that climate variables were conducive to bark beetle infestation across most of the Flat Tops study area. Relative to the entire Southern Rocky Mountains (inclusive of the Flat Tops), infestation in the Flat Tops was severely constrained by forest structure. The paucity of large diameter trees within the Flat Tops study area was a result of a severe spruce beetle outbreak that occurred 60 years ago. Stands infested during the 1940s in Flat Tops were notably depleted of large spruce relative to uninfested stands. Given the preference of spruce beetles for large diameter spruce and relative absence of large diameter spruce in areas affected by the 1940s infestation, it is not surprising that we found no overlap between areas of current infestation and areas affected by the 1940s infestation. These results support the hypothesis that stands affected by severe spruce beetle infestation are less susceptible to infestation c. 60 years later due to a decrease in large diameter spruce.

The 1940s spruce beetle infestation in northwestern Colorado was most severe in the Flat Tops area, where three-quarters of the 1940s spruce beetle-induced tree mortality occurred [32]. Nearby spruce-fir forests in Grand Mesa National Forest also experienced 1940s spruce beetle infestation, however it was significantly less severe [32,58]. For instance, the basal area of beetle-killed spruce was ca. 4—7.5x greater in the Flat Tops than in Grand Mesa [58]. In contrast, the 1997–2012 spruce beetle infestation has affected 2% of the spruce-fir forest in Flat Tops and 19% of Grand Mesa’s spruce-fir forest [34]. This suggests that the 1940s infestation in Grand Mesa was not severe enough to cause significant host depletion and thus Grand Mesa forests were much more susceptible to the 1997–2012 infestation.

Our study is notably limited by the availability of spatial datasets of both the 2000 and 1940s spruce beetle infestation. In particular we note that comparisons between these two datasets may be limited by the different methods used to map spruce beetle infestation (interpretation of aerial photography vs. aerial sketch mapping). However, our ability to accurately model the 1997–2012 infestation from a few ecologically meaningful biophysical predictors and the agreement between field data and maps of the 1940s outbreak suggest these datasets were appropriate for coarse assessment of the linkage between spruce beetle outbreaks. Subsequent analyses with datasets depicting severity of infestation at a fine spatial resolution would serve to advance our understanding of this linkage, however to our knowledge no such datasets exist for the Southern Rocky Mountains.

The findings of the current study indicate that at a broad spatial scale, severe spruce beetle outbreaks are linked disturbances (sensu [3]) at least over the 60-year period considered in our study. We suggest that the host depletion feedback not only may cause infestation collapse (sensu [32]), but may enhance ecological resistance (sensu [59]) of beetle-affected systems to spruce beetle infestation through long lasting effects of host depletion. Given that predictions of future beetle disturbance from climate-driven beetle population models do not incorporate process dynamics of disturbance-caused tree mortality and forest recovery [60], our results underscore the need for additional research on forecasting future forest dynamics, which may affect host availability for bark beetle infestations. In particular, the dampening effect of the 1940s spruce beetle infestation on the spread of the early 2000s infestation in the Southern Rocky Mountains implies that future infestations in the 21^st^ century may be similarly restricted by disturbance-caused depletion of susceptible hosts.

Most previous studies of linked disturbances in the coniferous forests of the Rocky Mountain region have addressed how previous fire affects subsequent bark beetle outbreaks [61,62] or how previous bark beetle outbreaks alters the probability, extent or severity of subsequent fire [5,15,20,36]. To our knowledge this is the first broad-scale analysis of how prior bark beetle outbreak affects susceptibility to subsequent bark beetle outbreak. Our findings of a dampening effect of the 1940s spruce beetle outbreak on susceptibility to spruce beetle infestation 60 years later highlights the need for incorporating the process dynamics of tree growth and mortality in predictive modeling of the likelihood of bark beetle outbreaks under future climate scenarios. Simulation modeling of the probability of future insect outbreaks based on climate suitability for the growth of the insect populations has been important in identifying likely trends over relatively short time periods. However, our results show that even at a time scale of 60 years, failure to incorporate negative feedbacks into prediction of future bark beetle outbreaks is likely to over-predict the extent or severity of future outbreaks and by implication under-estimate forest resistance to altered disturbance regimes under climate change.

## Supporting Information

S1 Fig Time series displaying the percentage of spruce-fir forest infested by spruce beetles. Data is shown for the Southern Rocky Mountain Ecoregion (inclusive of the Flat Tops) and only in the Flat Tops. For each region, the percent area was calculated by the determining the number of 990 x 990 m pixels within the spruce-fir zone identified as infested by the United States Forest Service in annual Aerial Detection Surveys (ADS) and dividing it by the total number of spruce-fir pixels.
(TIFF)

S2 Fig The importance of biophysical predictors in promoting spruce beetle infestation the Southern Rocky Mountain study region. Maps of (A) mean maximum August temperature (1997–2012), (B) mean annual precipitation (1997–2012), and (C) tree size class. The probability of infestation (derived from the classification tree in Fig 3B) is indicated by pixel color. Dark green indicates a probability of infestation <0.3, light green indicates a probability of infestation of 0.3–0.49, dark yellow indicates a probability of infestation 0.5–0.69, and dark brown indicates a probability of infestation >0.7. Sources are given in Table 1.
(TIFF)
