# Supplementary figures and images for: Negative Feedbacks on Bark Beetle Outbreaks: Widespread and Severe Spruce Beetle Infestation Restricts Subsequent Infestation
Source: PLoS One. 2015 May 22;10(5):e0127975. doi: 10.1371/journal.pone.0127975 (PMC4441381; doi:10.1371/journal.pone.0127975)

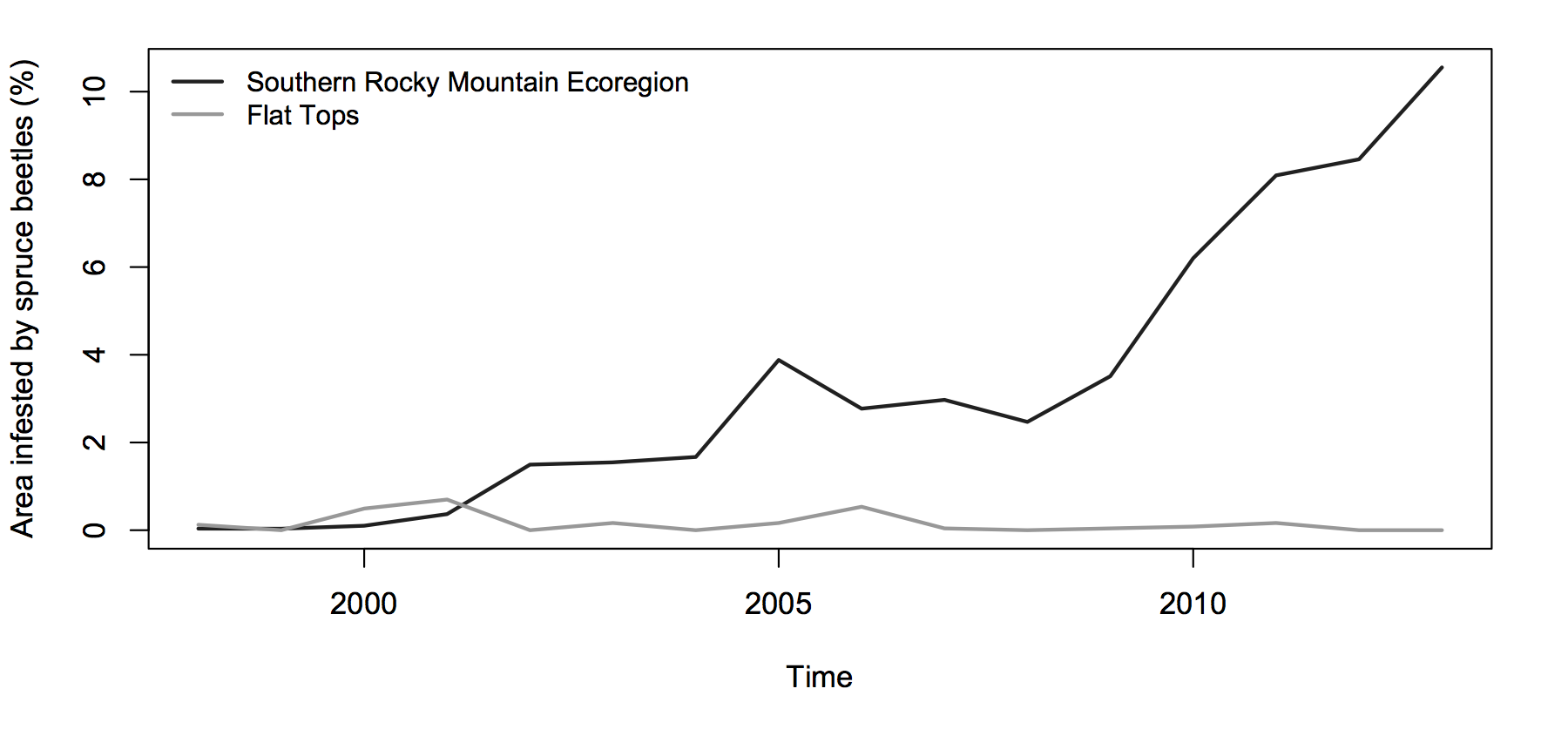

Supplement: S1 Fig — Data is shown for the Southern Rocky Mountain Ecoregion (inclusive of the Flat Tops) and only in the Flat Tops. For each region, the percent area was calculated by the determining the number of 990 x 990 m pixels within the spruce-fir zone identified as infested by the United States Forest Service in annual Aerial Detection Surveys (ADS) and dividing it by the total number of spruce-fir pixels. (TIFF) [file pone.0127975.s001.tiff]

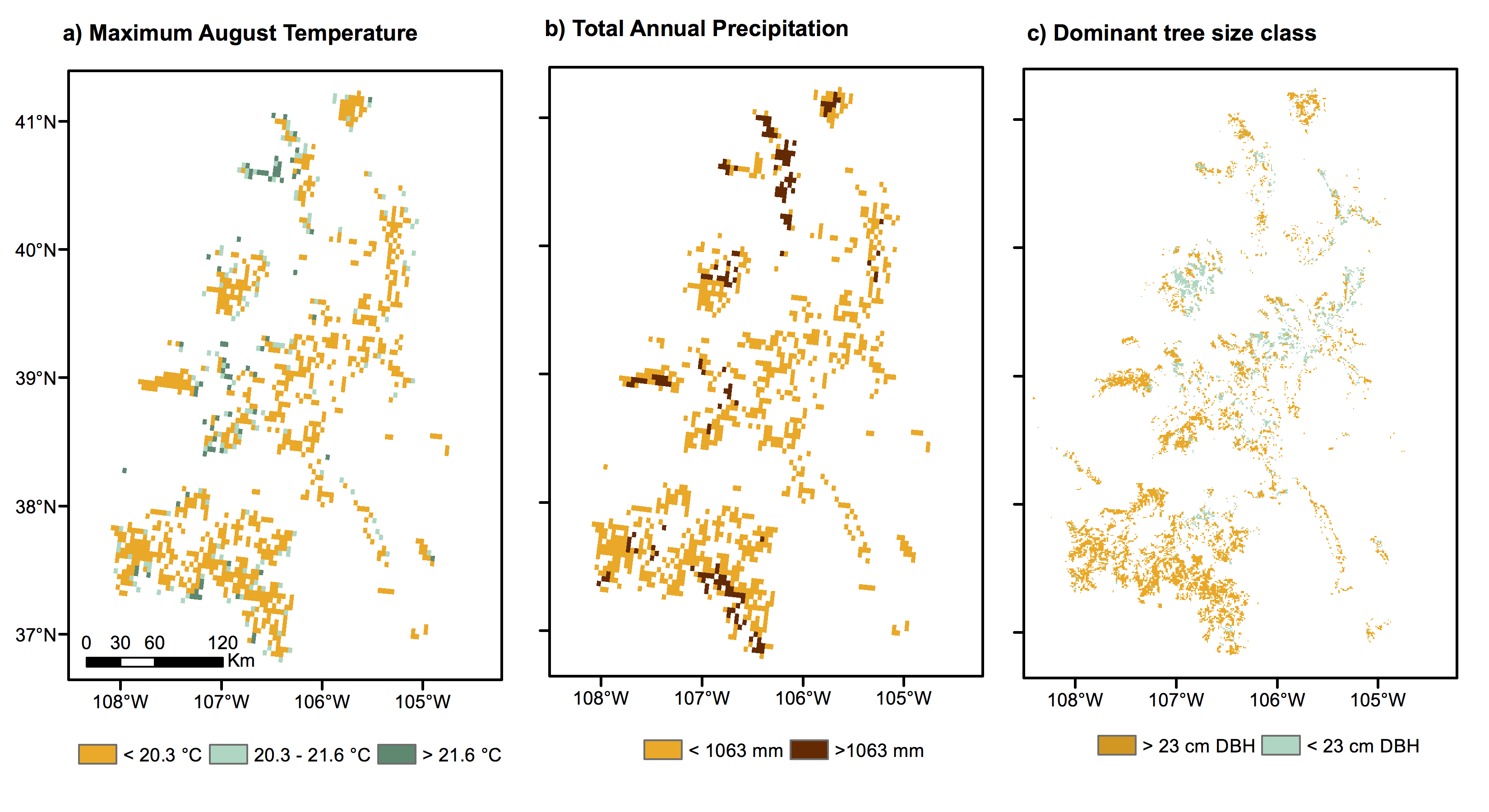

Supplement: S2 Fig — Maps of (A) mean maximum August temperature (1997–2012), (B) mean annual precipitation (1997–2012), and (C) tree size class. The probability of infestation (derived from the classification tree in Fig 3B) is indicated by pixel color. Dark green indicates a probability of infestation <0.3, light green indicates a probability of infestation of 0.3–0.49, dark yellow indicates a probability of infestation 0.5–0.69, and dark brown indicates a probability of infestation >0.7. Sources are given in Table 1. (TIFF) [file pone.0127975.s002.tiff]
